# Likelihood of Resident Doctors Raising Concerns Within an Acute Mental Health Trust

**DOI:** 10.1192/bjo.2025.10416

**Published:** 2025-06-20

**Authors:** Sarah O’Connor, Vicki Ibbett, Ruth Scally

**Affiliations:** Birmingham and Solihull Mental Health Foundation Trust, Birmingham, United Kingdom

## Abstract

**Aims:** Raising concerns is a vital component of optimising patient safety and improving training experiences. However, resident doctors within an acute mental health trust have expressed difficulties in raising such concerns. A quality improvement (QI) project was initiated to improve the self-reported likelihood of resident doctors raising patient safety and training concerns. We developed a pulse survey to capture this data and identify barriers to raising concerns, to thus inform and evaluate change ideas.

**Methods:** Over 17 months, a monthly pulse survey was distributed to all resident doctors within Birmingham and Solihull Mental Health Foundation Trust to ascertain their likelihood of raising both training and patient safety concerns using a Likert scale. Respondents were also asked to indicate the effectiveness of existing support systems for raising concerns. A free text box was available for respondents to detail other barriers or concerns. Demographic information was also collected and analysed. Data on the self-reported likelihood of raising concerns were plotted on run charts, with analysis in relation to the implementation of change ideas, to identify their effectiveness. Data on perceived barriers was utilised to inform change ideas.

**Results:** The mean survey response rate was 6.9 (range 1–21.4). Neither the likelihood of resident doctors raising concerns about patient.safety or training showed significant improvement, as evidenced by run charts. Barriers to raising concerns included the exception reporting system and feeling that no effective action would be taken. Residents were less likely to raise a concern if the severity was perceived as low. Over time the perception of the trust intranet as a supportive tool in raising concerns increased.

**Conclusion:** Although no significant changes were identified in resident doctors’ likelihood of raising concerns, the pulse survey provided valuable insight into what barriers still exist in our Trust for residents wanting to raise concerns. The feedback provided informed multiple change ideas that were implemented including modifications to the representative structure, improvements to the resident doctor meeting intranet page, and adaptations to the incident reporting system. Recommendations have also been made for future QI projects. The frequently changing workforce and low response rates meant quantitative data on the self-reported likelihood of raising concerns was limited. However, evaluation of specific change ideas showed a positive impact. Increased motivation of resident doctors to engage in QI projects was another noticeable achievement.

